# Supplementary figures and images for: Limiting DNA polymerase delta alters replication dynamics and leads to a dependence on checkpoint activation and recombination-mediated DNA repair
Source: PLoS Genet. 2021 Jan 25;17(1):e1009322. doi: 10.1371/journal.pgen.1009322 (PMC7861531; doi:10.1371/journal.pgen.1009322)

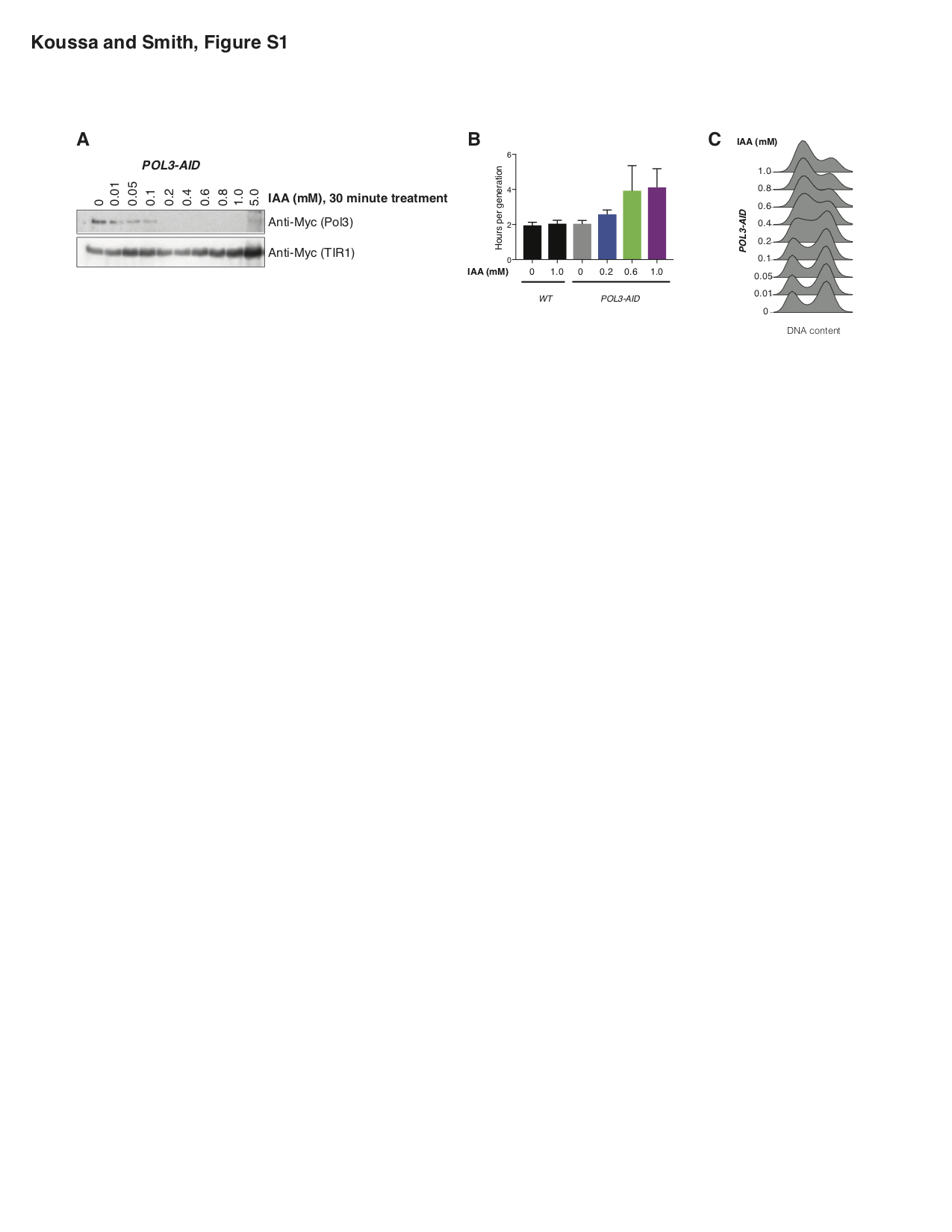

Supplement: S1 Fig — Further characterization of Pol3 depletion kinetics and the effect of depletion on growth rate. A. Western blot against Pol3-9Myc or OsTIR1-9Myc in asynchronous cultures of the POL3-AID strain following 30 minutes of treatment with the indicated concentration of IAA. B. Growth rates of POL3-AID cells in liquid culture. Data were calculated from three replicates. C. DNA content measured by flow cytometry for logarithmically growing POL3-AID cells treated with the indicated concentration of IAA for 2h. (TIFF) [file pgen.1009322.s001.tiff]

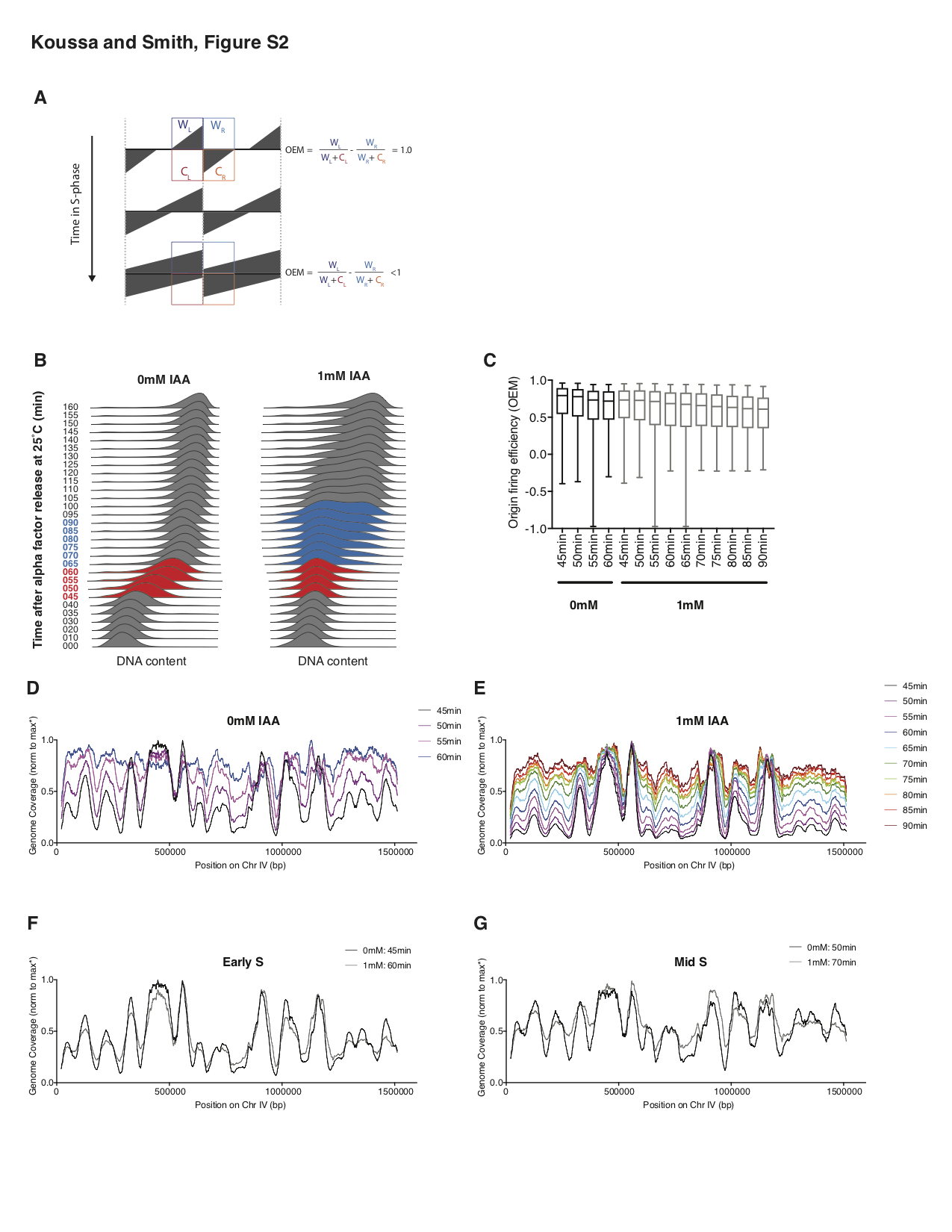

Supplement: S2 Fig — A. Schematic of expected Okazaki fragment distributions and OEMs for a moderately efficient replication origin (as calculated in [31]) over the course of S-phase. Okazaki fragments [grey] emanate from origins (dashed lines) to the left on the Watson strand (WL) or to the right on the Crick strand (CR). As S-phases progresses, the middle origin is passively replicated by the forks from the origins on the left and right in a fraction of cell, resulting in a lower OEM. B. Samples used for analysis of origin firing (Fig 2) and replication speed (Fig 4). DNA content measured by flow cytometry for the samples shown in Fig 4A. Red timepoints were sequenced for both 0 and 1 mM IAA, and blue timepoints for 1 mM only. C. Replication-origin firing efficiency for samples sequenced from S2A Fig, calculated as OEM from Okazaki fragment distributions around 281 high-confidence origins [31] for each sequenced time point across S phase. Data represent the mean efficiency averaged across two replicate strains at each time point. Whiskers indicate minimum and maximum. D-E. Total coverage of Okazaki fragment sequencing data across chromosome 4 for synchronous POL3-AID cultures treated with the indicated concentration of IAA for 2h and rapamycin for 1h treatment to deplete Cdc9 from the nucleus by anchor away. Data are normalized to the maximum of non-repetitive regions. F-G. Total coverage of Okazaki fragment sequencing data across chromosome 4 as in S2C and S2D Fig for matched timepoints in early (F) or mid (G) S phase. (TIFF) [file pgen.1009322.s002.tiff]

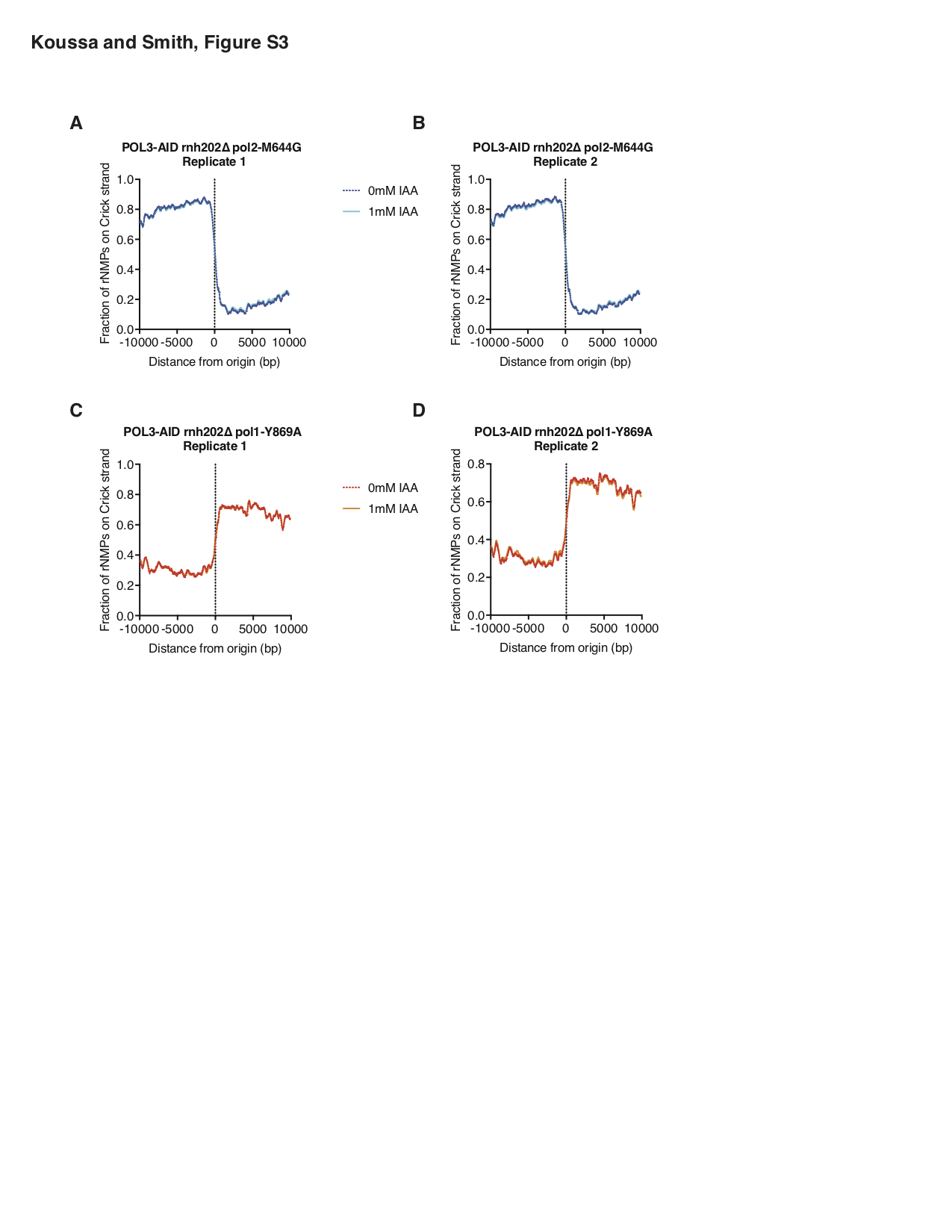

Supplement: S3 Fig — A-B. Analysis of ribonucleotide distribution around replication origins in the POL3-AID pol2- M644G rnh202Δ genetic background as shown for pooled replicates in Fig 3C, separated by individual biological replicate strain as indicated. C-D. Analysis of ribonucleotide distribution around replication origins in the POL3-AID pol1- Y869A rnh202Δ genetic background as shown for pooled replicates in Fig 3D, separated by individual biological replicate strain as indicated. (TIFF) [file pgen.1009322.s003.tiff]

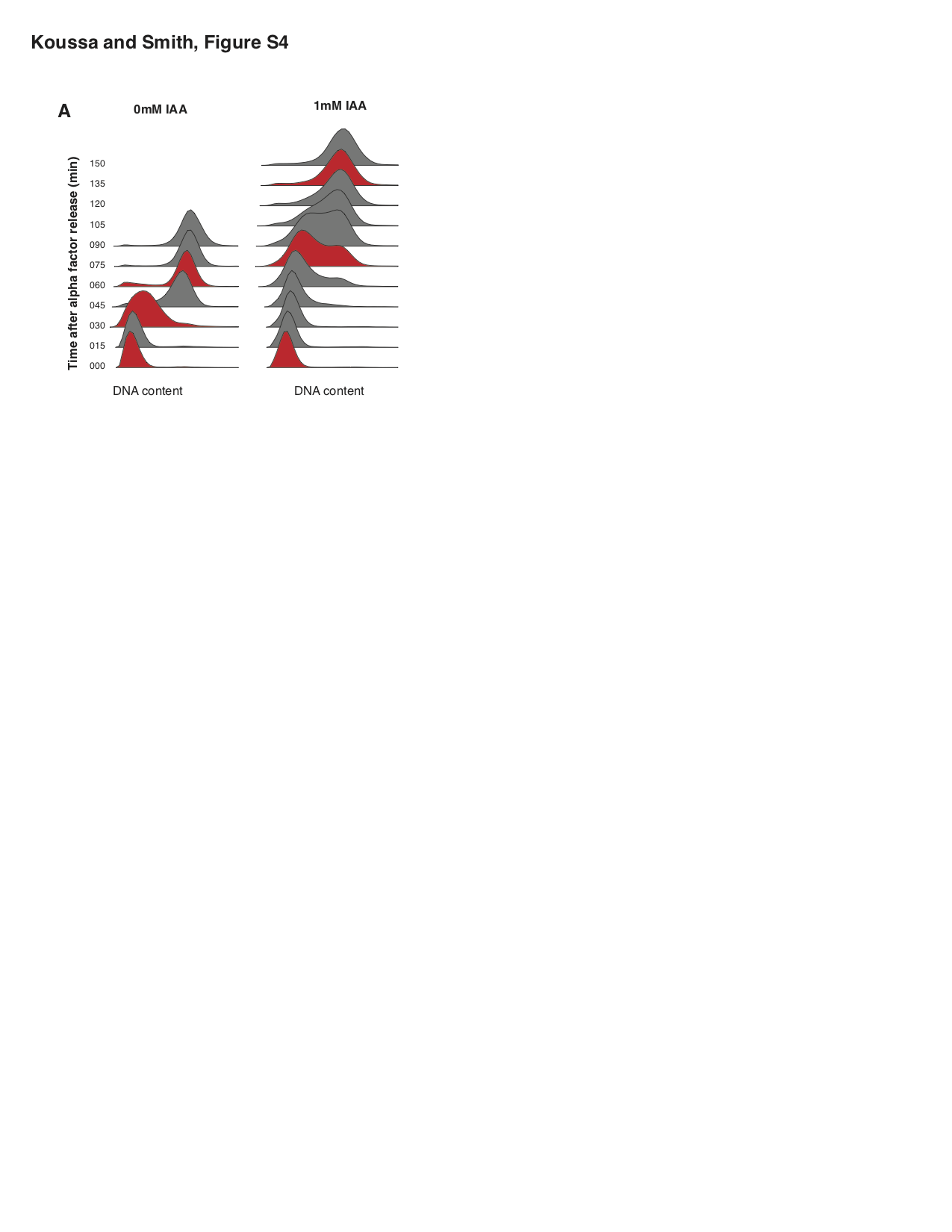

Supplement: S4 Fig — A. DNA content measured by flow cytometry for samples used for western blotting of Rfa1 in Fig 5B. Red timepoints were chosen for G1, mid S, or late S samples. (TIFF) [file pgen.1009322.s004.tiff]
